# The complete mitochondrial genome of *Meleonoma mirabilis* (Wang 2003) (Lepidoptera: Autostichidae)

**DOI:** 10.1080/23802359.2021.1944376

**Published:** 2021-08-01

**Authors:** Aihui Yin, Xin Yang

**Affiliations:** Morphological Laboratory, Guizhou University of Traditional Chinese Medicine, Guiyang, Guizhou, China

**Keywords:** *Meleonoma mirabilis*, mitochondrial genome, Autostichidae, phylogenetic analysis

## Abstract

The mitogenome of *Meleonoma mirabilis* was determined in this study. It was 15,268 bps long and strongly AT biased. It harbored 13 PCGs, 22 tRNAs, 2 rRNAs, and 1 non-coding control region (334 bps). BI phylogenetic analysis based on 13 concatenated PCGs from 24 moth species indicated that *M. mirabilis* was clustered in the family Autostichidae, which was consistent with the latest phylogenetic study.

*Meleonoma mirabilis* (Wang 2003), a small moth species, belongs to the genus *Meleonoma* Meyrick (Autostichidae, Lepidoptera), which had experienced substantial expansion in 2020. In that year, more than 100 species (most from China) were described as new or transferred from the other genus, and 8 species groups were set up in necessity to accommodate the rapid accumulation of new species, and to facilitate innergeneric taxonomy (e.g. Wang and Zhu 2020a, 2020b; Wang et al. 2020). Division of species groups was based on morphological analysis plus molecular evidence of one single *cox1* locus (Wang et al. 2020). More molecular loci should be involved to fully resolve this complex genus. Nevertheless, no mitogenome of *Meleonoma* was available so far. In compensation, we present herein the mitogenome of *M. mirabilis* which is a common species in South China (Wang et al. 2020). The adults were collected from Leigong Mountain Nature Reserve (26°22′23″‘N 108°11′52″‘E 1530 m), Guizhou, China in 2020, using light trap. The specimens were then deposited in 99% ethanol under −20 °C in the Insect Collection of Guizhou University of Traditional Chinese Medicine, Guiyang, China (Aihui Yin, keyanlaodong@163.com) under the voucher number GZUTCM:M59-62.

The genome sequencing was performed at Sangon Biotech (Shanghai) Co., Ltd., China, on Illumina HiSeq2500 platform. The de novo assembly was carried out with SPAdes V.3.14.1 (Bankevich et al. 2012) and NOVOPlasty V.4.0 (Dierckxsens et al. 2017). Gap filling and correctness check were executed manually with the aid of BWA V.0.7.17 (Li 2013), samtools V.0.1.19 (Li et al. 2009) and Pilon V.1.23 (Walker et al. 2014). MITOS WebServer (http://mitos2.bioinf.uni-leipzig.de/index.py) was utilized for annotation.

The double stranded circular mitogenome of *M. mirabilis* (GenBank: MW366996), was 15,268 bps long, and strongly AT biased (AT 77.1%, CG only 22.9%). It harbored the typical set of metazoan genes (13 PCGs, 22 tRNAs and two rRNAs) (Wolstenholme 1992), and one non-coding A + T rich control region (334 bps, AT 93.4%). Most PCGs of *M. mirabilis* started at conventional ATN start codon, only *cox1* initiated with CGA. All PCGs used the typical TAA or TAG stop codon at termination, except for *cox1*, *cox2*, *nad4*, and *nad5*, which terminated with an incomplete single T residue. Twenty-one out of 22 tRNAs were folded into the typical cloverleaf structure, leaving TrnS1 the only exception, as it lacked the DHU arm. Gene order TrnM-TrnI-TrnQ rather than the more ancestral non-Ditrysian trnI-trnQ-trnM was also recognized in *M. mirabilis* (Park et al. 2016).

The Bayesian inference analysis was conducted via MrBayes V.3.2.7 (Ronquist et al. 2012) using ‘GTR + I + G’ substitution model within the superfamily Gelechioidea. Thirteen concatenated PCGs from *M. mirabilis* and other 23 species from GenBank were used to rebuild the phylogenetic tree (Figure 1). Based on the currently available material, it showed that Autostichidae, Gelechiidae, Stathmopodidae, and Xyloryctidae which had multiple representatives were monophyletic. However, Oecophoridae was recovered as polyphyly. *M. mirabilis* was clustered in Autostichidae, which was consistent with the latest study (Wang and Li 2020).

**Figure 1.**
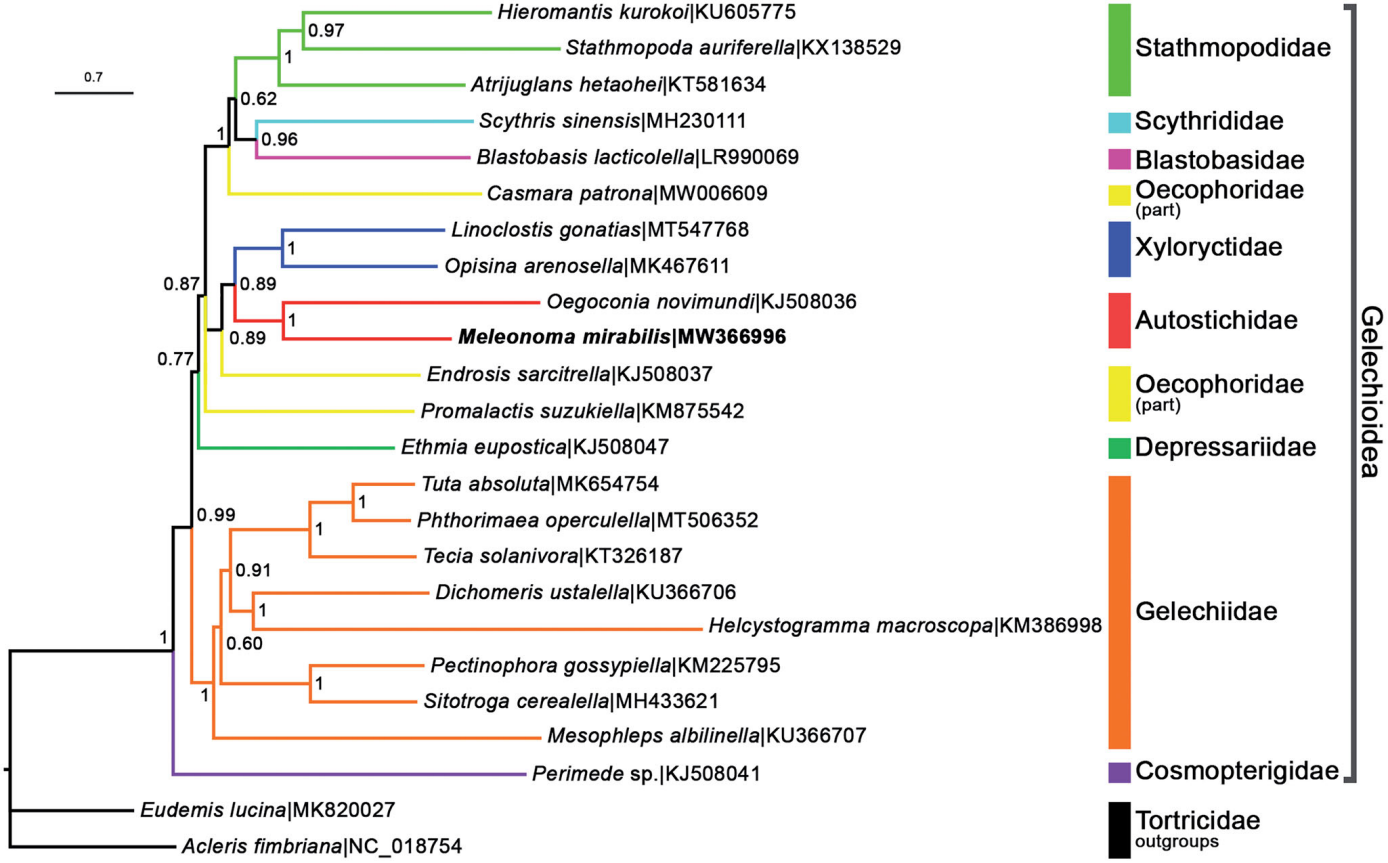
BI tree shows phylogenetic relationships among Gelechioidea moths. Bayesian posterior probabilities and GenBank accession numbers are indicated (*M. mirabilis* in bold type). Two representatives from Tortricidae were used as outgroups.

## Data Availability

The genome sequence data that support the findings of this study are openly available in GenBank of NCBI at https://www.ncbi.nlm.nih.gov/nuccore/MW366996 under the accession No. MW366996. The associated BioProject, SRA, and Bio-Sample numbers are PRJNA729565, SRR14517488, and SAMN19133288, respectively.
